# MiRNAs and piRNAs from bone marrow mesenchymal stem cell extracellular vesicles induce cell survival and inhibit cell differentiation of cord blood hematopoietic stem cells: a new insight in transplantation

**DOI:** 10.18632/oncotarget.6791

**Published:** 2015-12-29

**Authors:** Luciana De Luca, Stefania Trino, Ilaria Laurenzana, Vittorio Simeon, Giovanni Calice, Stefania Raimondo, Marina Podestà, Michele Santodirocco, Lazzaro Di Mauro, Francesco La Rocca, Antonella Caivano, Annalisa Morano, Francesco Frassoni, Daniela Cilloni, Luigi Del Vecchio, Pellegrino Musto

**Affiliations:** ^1^ Laboratory of Preclinical and Translational Research, IRCCS-Centro di Riferimento Oncologico della Basilicata (CROB), Rionero in Vulture, 85028, Italy; ^2^ Department of Clinical and Biological Sciences, University of Turin, Turin 10126, Italy; ^3^ Stem Cell Center, S. Martino Hospital, Genova 16132, Italy; ^4^ Transfusion Medicine Unit, Puglia Cord Blood Bank, IRCCS-Casa Sollievo della Sofferenza, San Giovanni Rotondo, 71013, Italy; ^5^ Laboratorio Cellule Staminali post natali e Terapie Cellulari, Giannina Gaslini Institute, Genova 16148, Italy; ^6^ CEINGE-Biotecnologie Avanzate S.C.a R.L., Naples, 80145, Italy; ^7^ Department of Molecular Medicine and Medical Biotechnologies, Federico II University, Naples 80131, Italy; ^8^ Scientific Direction, IRCCS-Centro di Riferimento Oncologico Basilicata (CROB), Rionero in Vulture, 85028, Italy

**Keywords:** mesenchymal stem cells, extracellular vesicles, umbilical cord blood stem cells, microRNAs, piRNAs

## Abstract

Hematopoietic stem cells (HSC), including umbilical cord blood CD34+ stem cells (UCB-CD34+), are used for the treatment of several diseases. Although different studies suggest that bone marrow mesenchymal stem cells (BM-MSC) support hematopoiesis, the exact mechanism remains unclear. Recently, extracellular vesicles (EVs) have been described as a novel avenue of cell communication, which may mediate BM-MSC effect on HSC. In this work, we studied the interaction between UCB-CD34+ cells and BM-MSC derived EVs. First, by sequencing EV derived miRNAs and piRNAs we found that EVs contain RNAs able to influence UCB-CD34+ cell fate. Accordingly, a gene expression profile of UCB-CD34+ cells treated with EVs, identified about 100 down-regulated genes among those targeted by EV-derived miRNAs and piRNAs (e.g. *miR-27b/MPL, miR-21/ANXA1, miR-181/EGR2*), indicating that EV content was able to modify gene expression profile of receiving cells. Moreover, we demonstrated that UCB-CD34+ cells, exposed to EVs, significantly changed different biological functions, becoming more viable and less differentiated. UCB-CD34+ gene expression profile also identified 103 up-regulated genes, most of them codifying for chemokines, cytokines and their receptors, involved in chemotaxis of different BM cells, an essential function of hematopoietic reconstitution. Finally, the exposure of UCB-CD34+ cells to EVs caused an increased expression CXCR4, paralleled by an *in vivo* augmented migration from peripheral blood to BM niche in NSG mice. This study demonstrates the existence of a powerful cross talk between BM-MSC and UCB-CD34+ cells, mediated by EVs, providing new insight in the biology of cord blood transplantation.

## INTRODUCTION

Umbilical cord blood CD34+ stem cells (UCB-CD34+) are a source of hematopoietic stem cells (HSC) used for the treatment of numerous diseases. Since HSC fate is bound to different factors, like bone marrow microenvironment, the communication between different cell types becomes an important mechanism of stemness maintenance and of differentiation [1]. In this *scenario*, cells communicate by growth factors, cytokines, adhesion molecules and extracellular vesicles (EVs) [2–4].

EVs are cell derived vesicles surrounded by a lipid bilayer, ranging from 30 nm to 2.000 nm in diameter depending on their origin [5]. EVs can be divided in three main classes: exosomes, microvecicles and apoptotic bodies, which are different in terms of size and mechanism of generation. [4–8]. EVs are enriched in phosphatidylserine, especially the budding ones, cytoplasmic protein, mRNA, miRNA and DNA [6, 9–11]. They mediate intercellular communication, interacting with target cells by controlling fundamental biological functions [4, 12, 13]. EVs have been isolated from different body fluids [14] and they have an important role not only in regulation of normal physiological processes [15] but also in several diseases [16–18]. Literature's data show that EVs are involved in tumor invasion, inflammation, blood clotting and, last but not least, regulation of stem cells [6, 19].

The maintenance of self-renewal, differentiation and aging of HSC can be influenced by bone marrow microenvironment [20, 21]. Since all the above-mentioned processes are strictly dependent and regulated by cellular communication, we studied the communication mediated by EVs.

Bone marrow mesenchymal stem cells (BM-MSC) are a component of hematopoietic microenvironment [22] and support hematopoiesis by the constitutive ability to secrete soluble factors as SCF, LIF, IL6 [23–25]. MSC are fibroblast-like multipotent cells present in different tissues, with the ability to differentiate into specialized cells like adipocytes, chondrocytes, osteoblasts, myocytes and neurons [26, 27]. In addition to their stem cell properties, MSC have been shown to possess immunoregolatory abilities influencing both adaptive and innate immune responses. Due to their ability, MSC and their derived EVs have a therapeutic effect in regenerative medicine [26–30], tumor growth/inhibition [31] and immunoregulation [13, 32].

On this basis, we isolated EVs from BM-MSC and characterized their small RNA cargo, that includes miRNA and piRNA. We found that BM-MSC-EVs influence UCB-CD34+ cell gene expression pattern, inducing cell survival, inhibiting cell differentiation and promoting their migration towards bone marrow.

Our findings, suggesting that BM-MSC-EVs could influence the UCB+CD34+ fate, may be helpful for their transplantation use.

## RESULTS

### Characterization of BM-MSC derived EVs

**Figure 1:**
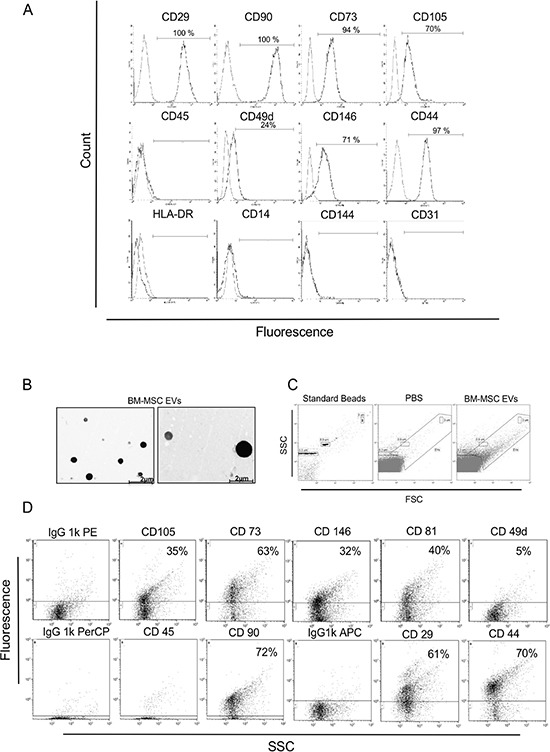
Immunophenotypic characterization of the BM-MSC and derived EVs (**A**) FITC, APC, PerCp and PE-conjugated antibodies were used for phenotyping the primary BM-MSC by flow cytometry. Cells were positive for CD29 (100%), CD90 (100%), CD73 (94%), CD105 (70%), CD49d (24%), CD146 (71%) and CD44 (97%) and negative for CD45, HLA-DR, CD14, CD144, CD31. One of three representative experiments is shown. (**B**) Two TEM representative images of EVs derived from BM-MSC are shown. EVs appear round and with heterogeneous size under 2 μm. (**C**) Representative FACS analysis of: 0,3 – 0,9 – 3 μm beads used as internal size standards, filtered PBS used like a negative control and BM-MSC-EVs. (**D**) Representative flow cytometric dot plots on BM-MSC derived vesicles showing positive BM-MSC markers (CD29, CD90, CD73, CD105, CD49d, CD146 and CD44), exosomes marker CD81 and negative hematopoietic marker CD45. Dot lines indicate the isotypic controls. The Kolmogrov-Smirnov statistical analyses between relevant antibodies and the isotypic control was significant (*P* < 0.001).

To characterize isolated BM-MSC, we checked the expression profile of MSC specific surface markers such as CD29, CD90, CD73, CD105, CD49d, CD146 and CD44. Cytometric analysis from three different experiments showed that cells were positive for CD29 (one representative experiment = 100%), CD90 (100%), CD73 (94%), CD105 (70%), CD49d (24%), CD146 (71%) and CD44 (97%) (Figure 1A). To confirm the purity of isolated BM-MSC, we also analyzed hematopoietic surface markers like HLA-DR, CD45, CD14 and endothelial marker like CD31 and CD144. In both cases they were negative (Figure 1A). EVs, isolated from BM-MSC, were detected by transmission electron microscopy showing spheroid morphology and a size lower than 2 μm (Figure 1B); moreover, flow cytometry analysis confirmed their size range between 100 nm and 2 μm (Figure 1C). EVs were positive, in two different experiments, for BM-MSC markers like CD29 (one representative experiment = 61%), CD90 (72%), CD73 (63%), CD105 (35%), CD146 (32%), CD44 (70%) and for exosome marker CD81 (40%); they were negative, instead, for hematopoietic marker CD45, proving their MSC cellular origin (Figure 1D).

### Identification of miRNA and piRNA expression signature in BM-MSC-EVs

**Figure 2:**
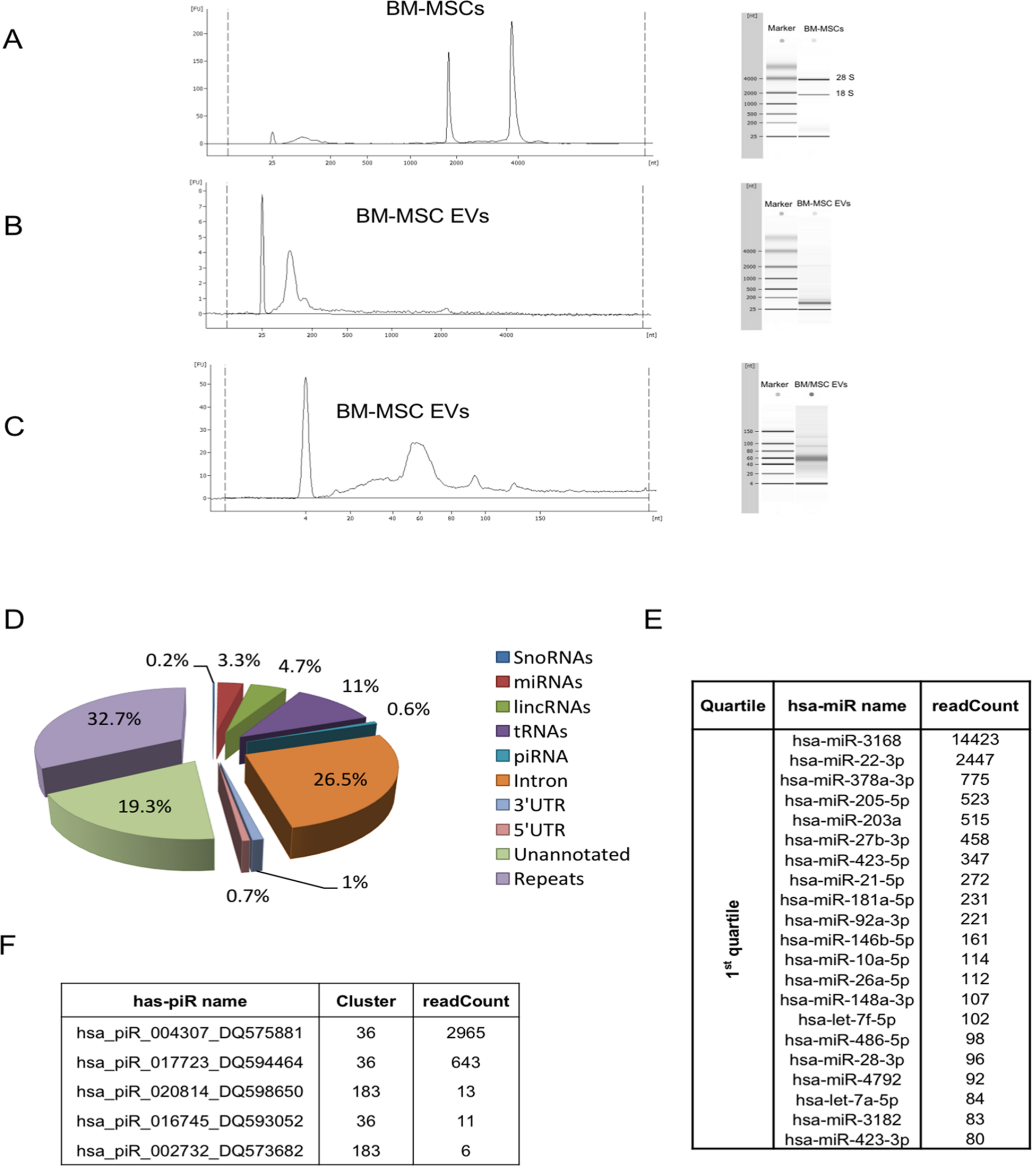
Bioanalyzer profile and small RNA sequencing of BM-MSC derived EVs Representative bioanalyzer profile of the RNAs contained in BM-MSC (**A**) and in BM-MSC-EVs (**B**). The electropherograms show the size distribution in nucleotides (nt) and fluorescence intensity (FU) of total RNA. The short peak at 25 nt is an internal standard. In BM-MSC the most dominant peaks are the 18S and 28S ribosomal RNA, whereas in EVs is the small RNAs peak respect to the absent rRNA peaks. (**C**) Representative bioanalyzer profile of small RNAs performed on EVs derived from BM-MSC showing an enrichment of small RNAs of the size of miRNAs in respect to the cells of origin. Three different samples of cells and EVs were analyzed. (**D**) Pie-charts showing the percentage of small RNA species identified in BM-MSC-EVs by small RNA sequencing. (**E**) Mature miRNA list obtained by sequencing BM-MSC-EVs small RNA content. The 87 microRNAs, obtained by miRanalyzer, were subdivided in quartiles on the base of readCount numbers and the most representative (1st quartile) were used in the integrated analysis with gene expression profile. (**F**) piRNA list obtained by sequencing BM-MSC-EVs small RNA content with readCount more than five.

A Bioanalyzer profile of total EVs RNAs revealed no peaks of ribosomal RNAs, 18S and 28S, and a characteristic peak of small RNAs below 200 bases in respect to origin cells (Figure 2A and 2B). Small-RNA analysis, conducted by a small-RNA chip assay, showed an enrichment of small-RNAs that fell within the range of 17–100 nucleotides (Figure 2C).

To characterize the miRNA and piRNA content in BM-MSC-EVs, we analyzed, by next generation sequencing, all small RNAs present in EVs. We obtained, after filtering out low quality reads and trimming the adaptor, about 1.5 million of reads. To identify the small-RNA distribution biotypes, we mapped all RNA reads to known RNA sequences and we found 3.3% of miRNAs and 0.6% of piRNAs sequences (Figure 2D).

From sequencing of data we identified 87 miRNAs by iMir tool [33], and 5 piRNAs with their clusters by piPipes tool [34] and piRNABank [35] (Figure 2E and 2F, Supplementary Material, Table S1).

### BM-MSC-EVs miRNA and piRNA sequencing and correlation with down-regulated genes in UCB-CD34+ cells treated with BM-MSC-EVs

**Figure 3:**
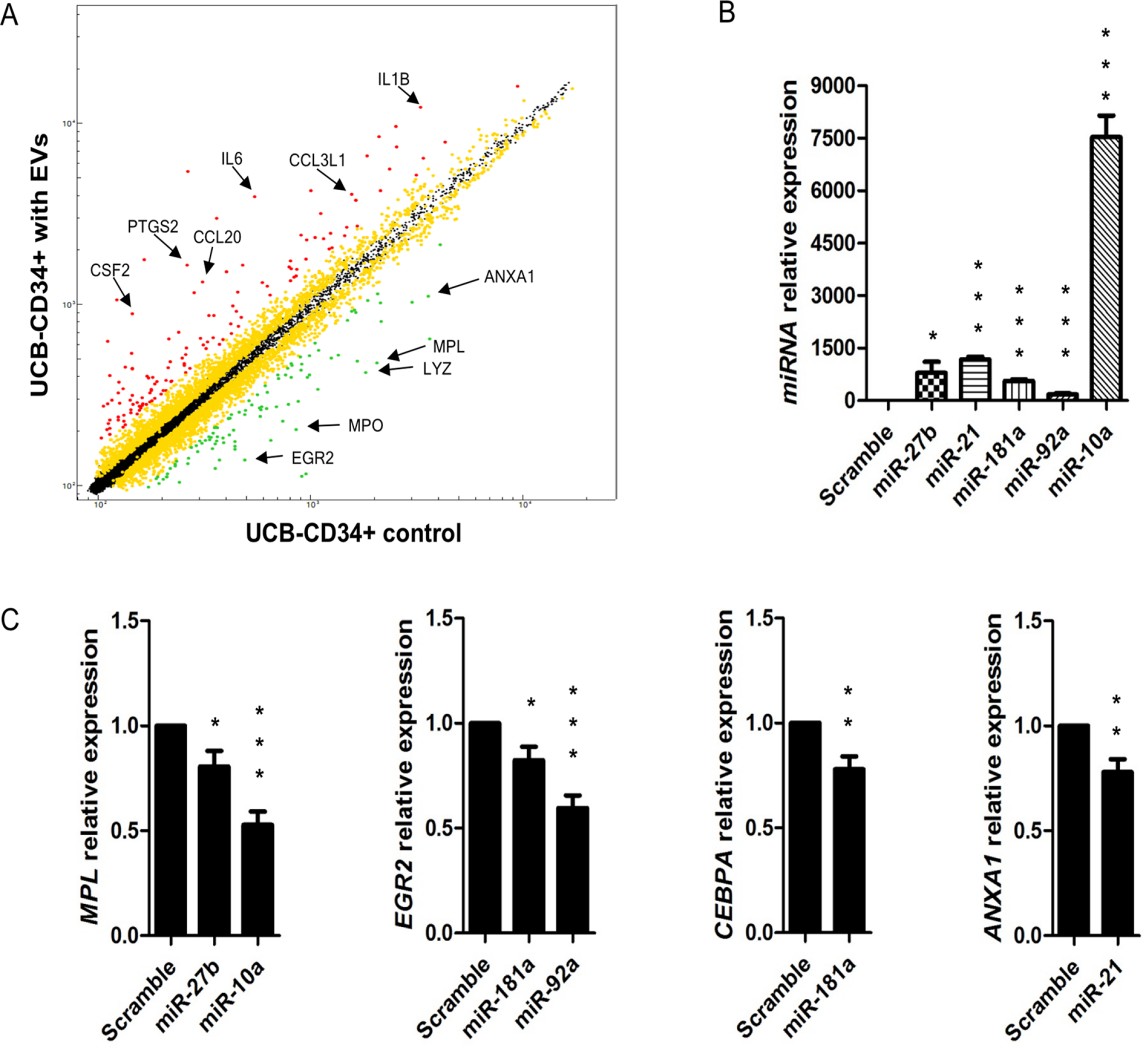
Gene expression profile of UCB treated with BM-MSC-EVs vs control and correlation with EVs miRNAs (**A**) Scatter plot of average gene expression levels between UCB-CD34+ treated with EVs and UCB-CD34+ control. Red and green dots represent up-regulated and down-regulated genes, respectively. Differential score was set at ±30 (*p* < 0.001) with log2fold difference ≤ −0.7 or ≥ 0.7 (more than 1.5-fold change). A list composed by 130 up-regulated and 100 down-regulated genes was used for further analysis. Yellow dots represent genes with a significant differential score (± 30; *p* < 0.001) but with a log2fold difference lower than 0.7 or upped than −0.7, and subsequently excluded for further analysis. With arrows are indicated the most representative genes in the Gene Ontology analysis with IPA software. (**B**) *miR-27b-3p, miR-10a-5p, miR-21-5p, miR-181a-5p and miR92a-3p* relative expression was determined by qRT-PCR in UCB-CD34+ transfected with 60 nM of miRNAs mimic *(miR-27b-3p, miR-10a-5p, miR-21-5p, miR-181a-5p and miR92a-3p)* and their scramble. Each data samples were normalized to the endogenous reference *RNU44* by use of the E^−ΔΔCp^. (**C**) *MPL, EGR2, CEBPA*, and *ANXA* mRNA relative expression was determined by qRT-PCR in UCB-CD34+ transfected with 60 nM of miRNAs mimic *(miR-27b-3p, miR-10a-5p, miR-21-5p, miR-181a-5p and miR92a-3p)* and their scramble. Each data samples were normalized to the endogenous reference *GAPDH* by use of the E^−ΔΔCp^. The bar-graphs represented mean + SD from three independent experiments. Statistically significant analyses are indicated by asterisks: *p < 0.05, **p < 0.01 and ***p < 0.001.

To define the changes due to BM-MSC-EVs miRNAs and piRNAs on gene expression patterns of UCB-CD34+ cells, we performed a gene expression profile comparing UCB-CD34+ cells treated with EVs versus not treated detecting 103 up-regulated and 100 down-regulated genes (with *p*-value < 0.05 and logFC = −0.7) (Figure 3A and Supplementary Material, Table S2). Interestingly, analyzing together the sequencing of EVs and gene expression data of UCB-CD34+ cells treated with EVs, by Ingenuity Pathways Analysis software, we identify at least one down-regulated target gene for each EVs miRNA (e.g., *miR-3168/LYZ*, *miR-27b-3p/ZFP36,miR21-5p/ANXA1*) (Table 1). To validate this correlation, we transfected five EVs miRNAs (*miR-27b-3p*, *miR-10a-5p*, *miR-21-5p*, *miR-181a-5p and miR92a-3p*) in UCB-CD34+ cells (Figure 3B) confirming the down-regulation of their predicted target genes (*miR-27b-3p/MPL and ZFP36*, *miR-10a-5p/MPL*, *miR-21-5p/ANXA1*, *miR-181a-5p/CEBPA* and *EGR2*, *miR92a-3p/ CEBPA* and *EGR2*) (Figure 3C).

**Table 1:** Correlation of the most representative (1st quartile) sequenced EVs miRNAs and down-regulated target genes in UCB-CD34+ treated with BM-MSC-EVs

miRNA		Regulated genes
*Name*	*readCount*	*Symbol (Entrez Gene ID)*
**hsa-miR-3168**	14423	**AGPAT9** (84803), **COL24A1** (255631), **LYZ** (4069)
**hsa-miR-22-3p**	2447	**AGPAT9** (84803), **CSF1R** (1436), **EMP1** (2012), **FCER2** (2208), **GPNMB** (10457), **KBTBD11** (9920), **RGS2** (5997)
**hsa-miR-378a-3p**	775	**FGR** (2268), **GPNMB** (10457), **ZFP36L2** (678)
**hsa-miR-205-5p**	523	**CAT** (847), **CYBB** (1536), **LHFPL2** (10184)
**hsa-miR-27b-3p**	458	**CYP1B1** (1545), **FOSB** (2354), **GLRX** (2745), **HOXA5** (3202), **KBTBD11** (9920), **MPL** (4352), **MTURN** (222166), **ZFP36** (7538), **ZFP36L2** (678)
**hsa-miR-423-5p**	347	**ALDH1A2** (8854), **C19orf59** (199675), **CLDN23** (137075), **CLEC5A** (23601), **FOSB** (2354), **MTURN** (222166), **ZFP36** (7538)
**hsa-miR-21-5p**	272	**ANXA1** (301), **OLR1** (4973), **S100A10** (6281), **TBC1D2** (55357), **TGFBI** (7045), **ZFP36L2** (678)
**hsa-miR-181a-5p**	231	**AHNAK** (79026), **CD163** (9332), **CDKN1B** (1027), **CEBPA** (1050), **CYP26B1** (56603), **DEPTOR** (64798), **EGR2** (1959), **FPR3** (2359), **MTURN** (222166), **SLA** (6503), **ZFP36L2** (678)
**hsa-miR-92a-3p**	221	**CEBPA** (1050), **CRHBP** (1393), **EGR2** (1959), **KLF2** (10365), **LHFPL2** (10184), **SGK1** (6446)
**hsa-miR-146b-5p**	161	**CSF1R** (1436), **PPBP** (5473), **SDCBP** (6386), **TBC1D2** (55357)
**hsa-miR-10a-5p**	114	**CLEC5A** (23601), **KBTBD11** (9920), **MPL** (4352)
**hsa-miR-26a-5p**	112	**HOXA5** (3202), **HOXA9** (3205)
**hsa-miR-148a-3p**	107	**CDKN1B** (1027), **CLEC5A** (23601), **CTSL** (1514), **EMP1** (2012), **FOSB** (2354), **GLIPR1** (11010), **LIPA** (3988), **MPL** (4352)
**hsa-miR-486-5p**	98	**EMP1** (2012)
**hsa-miR-28-3p**	96	**COL24A1** (255631)
**hsa-miR-4792**	92	**ACP5** (54), **RGCC** (28984)
**hsa-let-7a-5p**	84	**AGPAT9** (84803), **CCL7** (6354), **COL24A1** (255631), **EVI2B** (2124), **GLRX** (2745), **HOXA9** (3205), **MYCN** (4613), **OLR1** (4973), **PLD3** (23646)
**hsa-miR-3182**	83	**CYBB** (1536), **IGFBP7** (3490)
**hsa-miR-423-3p**	80	**ZFP36L2** (678)

Along with miRNAs, recent studies indicated that also piRNAs play a role, not only in transposon silencing, but also in epigenetic and in post-transcriptional regulation of gene expression [36, 37]. Based on this evidence, we postulate that piRNAs presence could influence mRNAs expression in UCB-CD34+ treated with EVs. To identify the target mRNAs of our piRNAs, we used miRanda matching each piRNA sequence with 5′-UTRs, CDSs or 3′-UTRs regions of all known human RNAs databases. Surprisingly, we found from the down-regulated genes of our profile only 4 putative mRNA target sequences of two piRNAs *(hsa_piR_020814_DQ598650, hsa_piR_002732_DQ573682)* (Table 2). To identify the target mRNAs for the other 3 piRNAs *(hsa_piR_004307_DQ575881, hsa_piR_017723_DQ594464, hsa_piR_016745_DQ593052)* on our gene expression analysis, we modified the logFC form −0.7 to −0.4 detecting a wider list of about 400 down-regulated genes. Subsequently, analyzing together all piRNA target sequences and the down-regulated genes, by IPA, we identify 18 piRNA target genes (Supplementary Material, Table S3). These data indicate that the EVs miRNAs and piRNAs are able to modify gene expression profile of receiving cells.

**Table 2:** Correlation of sequenced EVs piRNAs and down-regulated target genes in UCB-CD34+ treated with BM-MSC-EVs (*p*-value < 0.05, logFC = −0.7)

miRNA		Regulated genes
*Name*	*readCount*	*Symbol (Entrez Gene ID)*
hsa_piR_020814_DQ598650	13	**MPO** (4353), **SLC2A5** (6518), **SLAMF8** (56833)
hsa_piR_002732_DQ573682	6	**CYP1B1** (1545)

### EVs treatment induces cell survival and inhibits apoptosis of UCB-CD34+ cells

**Figure 4:**
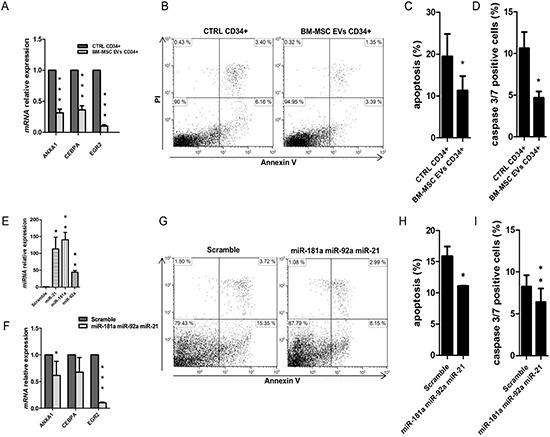
Apoptosis analysis in UCB-CD34+ treated with BM-MSC-EVs vs control (**A**) *ANXA1, CEBPA* and *EGR2* mRNA relative expression was determined by qRT-PCR in UCB-CD34+ treated with 20 μg/ml of BM-MSC-EVs for 24 hours and their control. Each data samples were normalized to the endogenous reference *GAPDH* by use of the E^−ΔΔCp^. (**B**) Dot plot of one experiment shows a double PI/annexin V staining of UCB-CD34+ after 24 h of co-culture with or without BM-MSC-EVs (**C**) The mean percentage of apoptotic UCB-CD34+, after 24 h of co-culture with or without BM-MSC-EVs, was evaluated by PI/annexin V test. Results are expressed as a mean + SD of four independent experiments. (**D**) The percentage of caspase 3/7 positive UCB-CD34+, after 24 h of co-culture with or without BM-MSC-EVs, was evaluated by Caspase-3/7 Green flow cytometry assay. (**E**) *miR-21-5p, miR-181a-5p and miR92a-3p* relative expression was determined by qRT-PCR in UCB-CD34+ co-transfected with miRNAs mimic *(miR-21-5p, miR-181a-5p* and *miR92a-3p)* and scramble. Each data samples were normalized to the endogenous reference *RNU44* by use of the E^−ΔΔCp^. (**F**) *EGR2* and *ANXA1* mRNA relative expression was determined by qRT-PCR in UCB-CD34+ co-transfected with miRNAs mimic and their scramble. Each data samples were normalized to the endogenous reference *GAPDH* by use of the E^−ΔΔCp^. (**G**) Representative image showing a double PI/annexin V staining of UCB-CD34+ after 24 h of miRNAs mimic or scramble transfection. (**H**) The percentage of apoptotic UCB-CD34+, after 24 h of miRNAs mimic or scramble transfection, was evaluated by PI/annexin V test. (**I**) The percentage of caspase 3/7 positive UCB-CD34+, after 24 h of miRNAs mimic or scramble transfection, was evaluated by Caspase-3/7 Green flow cytometry assay. Results are expressed as a mean + SD of three independent experiments. Statistically significant analyses are indicated by asterisks: *p < 0.05, **p < 0.01 and ***p < 0.001.

To characterize cell biological processes modulated by EVs miRNAs, we analyzed miRNA-targeted genes in UCB-CD34+ cells by gene expression profile. Gene ontology analysis revealed that all genes were involved in different down-regulated functions as cell death, growth and proliferation, movement and development (Table 3). Cell death was the first and the most down-regulated function (*p* = 3.89^−10^) identified from the analysis, including about 35 miRNA-targeted genes from the profile (Table 4). To validate gene expression analysis, we confirmed, by real Time PCR, the down-regulation of some genes like *ANXA1*, *CEBPA* and *EGR2* (*p*-value < 0.001) respect to the control (Figure 4A). Of interest, similarly to miRNA also piRNA-targeted genes from our profile (with *p*-value < 0.05 and logFC = −0.4) resulted to be involved in the previous listed down-regulated function as cell death (Supplementary Material, Table S4).

**Table 3:** Molecular and cellular functions of down-regulated genes in UCB-CD34+ treated with BM-MSC-EVs vs control (*p*-value < 0.05, logFC = −0.7)

Gene Ontology Bio Functions	*p*-value
***Molecular and Cellular Functions***	
**Cell Death and Survival**	3.89^−10^–4.07^−03^
**Cell Morphology**	2.20^−09^–4.07^−03^
**Cellular Growth and Proliferation**	2.20^−09^–3.85^−04^
**Cellular Movement**	4.42^−08^–4.15^−03^
**Cellular Development**	1.22^−07^–4.29^−03^

**Table 4:** Down-regulated miRNA target genes involved in cell death

Functions	*p*-value	Molecules
**cell death**	3.89E–10	CDKN1B,CEBPA,ALDH1A2,ANXA1,CAT,CCL7,CLEC5A,CSF1R,CTSL,CYBB,CYP1B1,CYP26B1,DEPTOR, EGR2,EMP1,FCER2,FGR,FOSB,GLIPR1,GLRX,HOXA5,HOXA9,IGFBP7,KLF2,LYZ,MPL,MYCN,OLR1, PPBP,S100A10,SGK1,TGFBI,ZFP36,ZFP36L2
**apoptosis**	7.43E–10	CDKN1B,CEBPA,ALDH1A2,ANXA1,CAT,CLEC5A,CSF1R,CTSL,CYBB,CYP1B1,CYP26B1,DEPTOR,EGR2, FCER2,FGR,FOSB,GLIPR1,GLRX,HOXA5,HOXA9,IGFBP7,KLF2,MPL,MYCN,OLR1,S100A10,SGK1, TGFBI,ZFP36,ZFP36L2
**necrosis**	5.88E–08	CDKN1B,CEBPA,ALDH1A2,ANXA1,CAT,CSF1R,CYBB,CYP1B1,DEPTOR,EGR2,EMP1,FCER2,FGR,FOSB, GLIPR1,GLRX,HOXA5,HOXA9,IGFBP7,KLF2,MPL,MYCN,OLR1,PPBP,S100A10,SGK1,ZFP36

Moreover, we demonstrated that BM-MSC EVs are able to significantly inhibit apoptotic pathways (*p*-value < 0.05) (Figure 4B and 4C) and caspase 3/7 activity (*p*-value < 0.05) (Figure 4D) in UCB-CD34+ cells compared to the control.

No difference, instead, was found between UCB-CD34+ cells cultured in conditioned BM-MSC medium deprived of EVs or in medium alone (data not shown), indicating that BM-MSC-EVs carried specific miRNAs to make cells more viable.

To further confirm apoptosis process reduction due to BM-MSC-derived EVs miRNAs, we transfected together *miR-21-5p*, *miR-181a-5p and miR92a-3p* (Figure 4E) in UCB-CD34+. After transfection we found *ANXA1*, *CEBPA* and *EGR2*, significantly down-regulated (*ANXA1 p*-value < 0.05 and *EGR2 p*-value < 0.001) (Figure 4F) in transfected UCB-CD34+ cells vs control. Furthermore, the same cells showed a significant decrease of apoptosis pathway (*p*-value < 0.05) (Figure 4G and 4H) and of caspase 3/7 activity (*p*-value < 0.05) (Figure 4I).

### EVs treatment induces a decrease of differentiation of UCB-CD34+ cells

**Figure 5:**
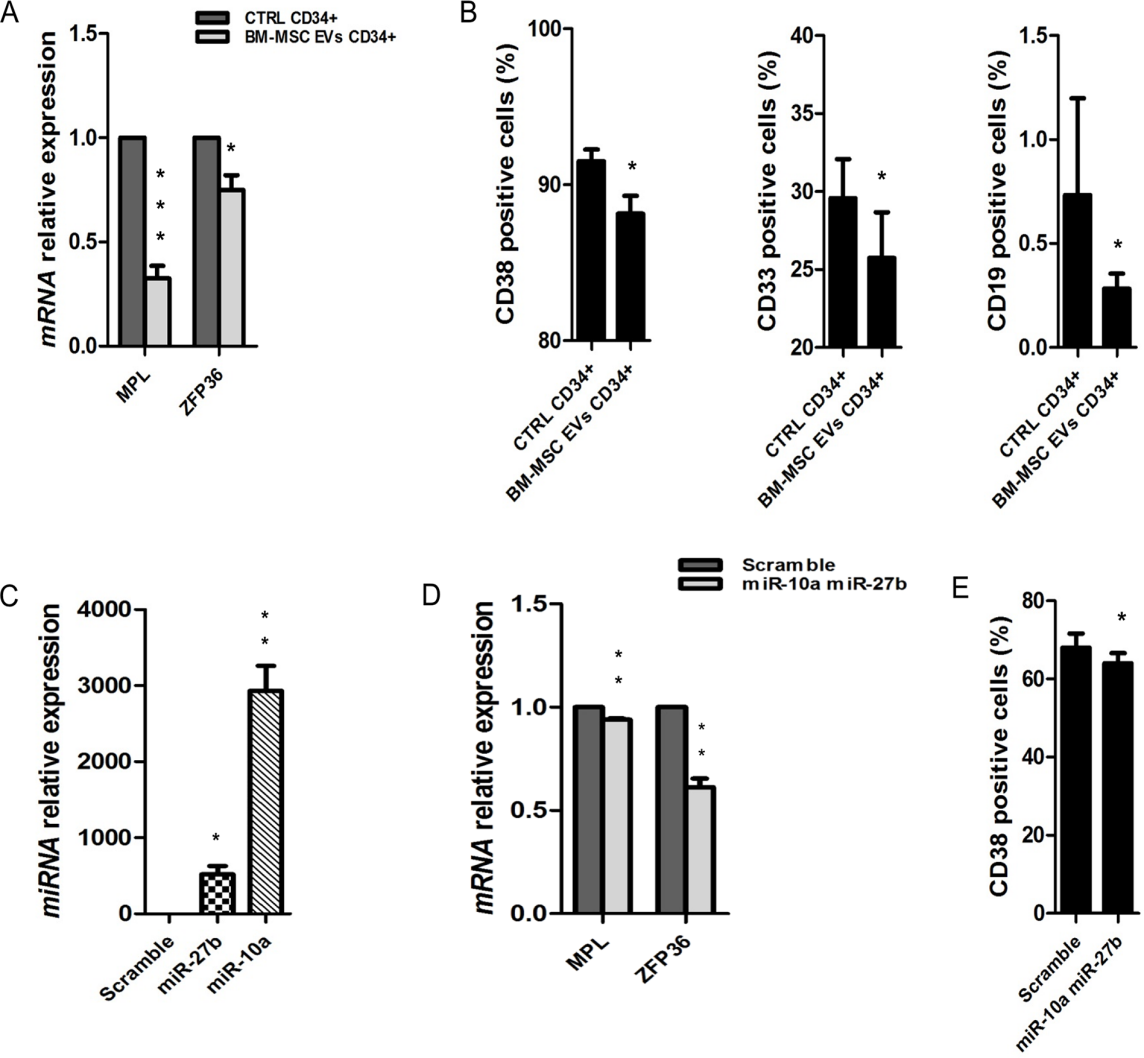
Cellular differentiation in UCB-CD34+ treated with BM-MSC-EVs vs control (**A**) *MPL* and *ZFP36* mRNA relative expression was determined by qRT-PCR in UCB-CD34+ treated with 20 μg/ml of BM-MSC-EVs for 24 hours and their control. Each data samples were normalized to the endogenous reference *GAPDH* by use of the E^−ΔΔCp^. (**B**) The percentage of CD38, CD33 and CD19 positive UCB-CD34+, after 24 h of co-culture with or without BM-MSC-EVs, was evaluated by FACS analysis. (**C**) *miR-27b-3p* and *miR-10a-5p* relative expression was determined by qRT-PCR in UCB-CD34+ co-transfected with miRNAs mimic (*miR-27b-3p* and *miR-10a-5p*) and scramble. Each data samples were normalized to the endogenous reference *RNU44* by use of the E^−ΔΔCp^. (**D**) *MPL* mRNA relative expression was determined by qRT-PCR in UCB-CD34+ co-transfected with miRNAs mimic and their scramble. Each data samples were normalized to the endogenous reference *GAPDH* by use of the E^−ΔΔCp^. (**E**) The percentage of CD38 positive UCB-CD34+, after transfection of miRNAs mimic or scramble, was evaluated by FACS analysis. Results are expressed as a mean + SD of three independent experiments. Statistically significant analyses are indicated by asterisks: *p < 0.05, **p < 0.01 and ***p < 0.001.

EVs miRNAs induce a decrease of UCB-CD34+ cells differentiation. Analyzing the 16 genes identified in this class of function from the gene expression profile, we observed a decrease of RNAs involved in cellular maturation to all hematopoietic lineages (Table 5). To validate the down-regulation of these genes, we assessed the transcript levels of some of them like *MPL* and *ZFP36*. As expected, both genes were significantly down-regulated (*MPL p*-value < 0.001 and *ZFP36 p*-value = 0.02) in UCB-CD34+ cells treated with EVs respect to the control (Figure 5A).

**Table 5:** Down-regulated miRNA target genes involved in cell development and differentiation

Functions	*p*-value	Molecules
**development of blood cells**	5.21E–05	ANXA1,CDKN1B,CEBPA,CSF1R,EGR2,HOXA5,HOXA9,KLF2,MPL,SLA
**differentiation of leukocytes**	5.48E–05	ANXA1,CDKN1B,CEBPA,CSF1R,EGR2,HOXA5,HOXA9,MYCN,SLA,ZFP36
**differentiation of myeloid cells**	1.73E–04	CDKN1B,CEBPA,CSF1R,HOXA5,HOXA9,ZFP36
**differentiation of myeloid progenitor cells**	3.12E–04	CDKN1B,CEBPA,HOXA9
**differentiation of mononuclear leukocytes**	3.39E–04	ANXA1,CDKN1B,CEBPA,CSF1R,EGR2,HOXA9,MYCN,SLA
**development of leukocytes**	6.80E–04	ANXA1,CDKN1B,CEBPA,CSF1R,EGR2,HOXA9,KLF2,SLA
**differentiation of hematopoietic progenitor cells**	7.95E–04	CDKN1B,CEBPA,CSF1R,HOXA5,HOXA9
**differentiation of cells**	8.91E–04	ALDH1A2,ANXA1,CAT,CDKN1B,CEBPA,CSF1R,CYP26B1,EGR2,HOXA5, HOXA9,IGFBP7,MPL,MYCN,RGS2,SLA,ZFP36
**development of hematopoietic progenitor cells**	1.99E–03	CDKN1B,EGR2,HOXA5,MPL
**development of lymphocytes**	2.17E–03	ANXA1,CDKN1B,CEBPA,EGR2,HOXA9,KLF2,SLA
**development of bone marrow cells**	2.22E–03	CEBPA,HOXA5,MPL
**differentiation of granulocytes**	3.00E–03	CDKN1B,CEBPA,HOXA9
**differentiation of monocytes**	3.16E–03	CEBPA,CSF1R,HOXA9
**differentiation of neutrophils**	4.29E–03	CEBPA,HOXA9

A reduction of cell differentiation is also one of the most down-regulated function found by the analysis of piRNA down-regulated target genes (Supplementary Material, Table S4) supporting the idea that EVs miRNAs and piRNAs regulate the same functions in UCB-CD34+ cells.

Moreover, a decrease of cellular maturation was confirmed by FACS analysis, evaluating different hematological markers (CD19+, CD33+ and CD38+). The expression of CD19+, CD33+ and CD38+ resulted to be significantly lower in UCB-CD34+ cells treated with EVs when compared to control (CD19+ and CD33+: *p*-value < 0.05; CD38+: *p*-value < 0.01) (Figure 5B). No difference, instead, was found in UCB-CD34+ cells cultured in conditioned BM-MSC medium deprived of EVs compared to UCB-CD34+ cells cultured in medium alone (data not shown). To demonstrate that a decrease of cell differentiation is induced by BM-MSC EVs miRNAs we transfect together *miR-27b-3p* and *miR-10a-5p* (Figure 5C). After transfection we found significantly reduced the expression of *MPL* and *ZFP36* (*p*-value < 0.01) (Figure 5D) and of the hematological marker CD38+ (*p*-value < 0.05) (Figure 5E) in transfected UCB-CD34+ cells vs control.

### EVs treatment induces the expression of chemotactic factors from UCB-CD34+ cells

**Figure 6:**
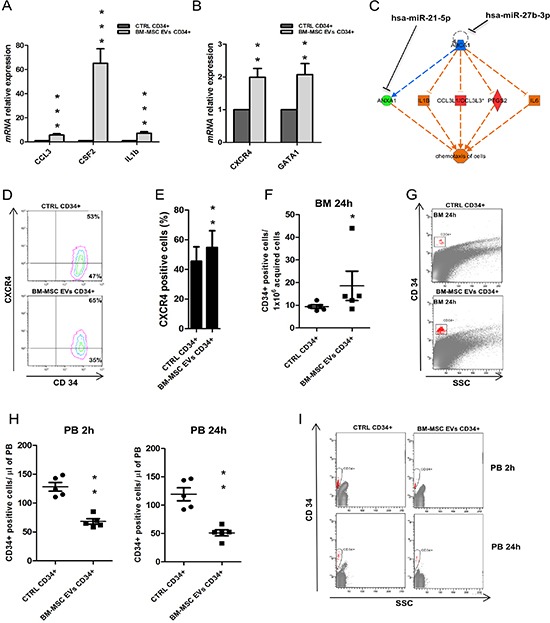
Expression of chemotactic factors in UCB-CD34+ treated with BM-MSC-EVs vs control (**A**–**B**) *IL1b*, *CSF2*, *CCL3*, *GATA2* and *CXCR4* mRNA relative expression was determined by qRT-PCR in UCB-CD34+ treated with 20 μg/ml of BM-MSC-EVs for 24 hours and their control. Each data samples were normalized to the endogenous reference *GAPDH* by use of the E^−ΔΔCp^. (**C**) *In-silico* regulator effect analysis of EVs sequenced miRNAs and UCB-CD34+ gene expression profile treated with vesicles. Chemotaxis of cells is supported by the up-regulation of selected genes (*IL1B, CCL3L1/3, PTGS2, IL6, IL23A, CFS2*) that are under control of down-regulated genes (*ZFP36, ABCA1, ANXA1*). All these genes are differentially expressed in GEP analysis. Down-regulated genes are *in-silico* linked (by TargetScan) to most representative miRNA sequenced by EVs. (**D**) Representative image showing a double anti-CD34/anti-CXCR4 staining of UCB-CD34+ after 24 h of co-culture with or without BM-MSC-EVs. (**E**) The percentage of CXCR4 positive UCB-CD34+, after 24 h of co-culture with or without BM-MSC-EVs, was evaluated by FACS analysis. (**F**) Number of human CD34 positive cells per 1 × 10^6^ acquired cells in murine BM after 24 hours of transplantation. (**G**) Representative image of F. (**H**) Number of human CD34 positive cells per μl of murine PB acquired, after 2 and 24 hours of transplantation. (**I**) Representative image of H. The Results are expressed as a mean + SD of three independent experiments. Statistically significant analyses are indicated by asterisks: *p < 0.05, **p < 0.01 and ***p < 0.001.

Through gene expression profile, we identified 103 up-regulated genes. To validate their over-expression, we evaluated the mRNA expression levels of some of them, like *IL1b, CSF2, CCL3, GATA2* and *CXCR4*. The transcript levels of all genes resulted significantly up-regulated (*IL1b, CSF2* and *CCL3*: *p*-value < 0.001; *GATA2* and *CXCR4*: *p*-value < 0.01) on UCB-CD34+ cells treated with EVs respect to the control (Figure 6A and 6B).

Furthermore, using IPA, we found that some of them (e.g., IL1b, CSF2, and CCL3) are under the control of genes targeted by miRNAs present in EVs (e.g., *ZFP36/miR-27b-3p* and *miR-423-5p, ABCA1/ miR-27b-3p*) (Figure 6C). This analysis allowed to identify correlation pathways between up- and down-regulated genes driven by EVs miRNAs and having like a target function the chemotaxis of BM microenvironment cells.

Moreover, gene ontology analysis of up-regulated genes classified cellular movement and interaction from the most activated functions (Table 6). C-X-C chemokine receptor type 4 (CXCR4), for example, is a physiologic receptor of CXCL12 expressed on HSC and essential for the engraftment of CD34+ cells in BM niche. CXCL12/CXCR4 interaction is a key component of HSC niche and their signal is necessary for maintaining HSCs quiescent [38, 39]. We evaluated CXCR4 expression observing its significant increase in UCB-CD34+ after EVs treatment respect to the control (*p*-value < 0.01) (Figure 6D and 6E). In light of these results, we investigated *in vivo* migration of UCB-CD34+ cells after EVs treatment. Treated cells demonstrated a significant increased engraftment (*p*-value < 0.01) of human cells in murine BM when compared to control cells (Figure 6F and 6G). Moreover, a significant decrease, at 2 hours (*p*-value < 0.01) and 24 hours (*p*-value < 0.01) after injection, was observed between the number of peripheral blood human cells in mice engrafted with UCB-CD34+ treated versus control (Figure 6H–6I).

**Table 6:** Molecular and cellular functions of up-regulated genes in UCB-CD34+ treated with BM-MSC-EVs vs control

Gene Ontology Bio Functions	*p*-value
***Molecular and Cellular Functions***	
**Cellular Movement**	2.25^−39^–2.42^−07^
**Cell-To-Cell Signaling and Interaction**	8.73^−30^–2.28^−07^
**Cellular Function and Maintenance**	1.82^−28^–3.37^−08^
**Cellular Growth and Proliferation**	2.31^−27^–2.51^−07^
**Cellular Development**	6.09^−27^–1.22^−07^

This analysis indicate that the EVs miRNAs induce, in UCB-CD34+ cells, the expression of different factors and receptors able to stimulate communication and movement of stem cells towards the niche microenvironment.

## DISCUSSION

Cell communication represents a dynamic mechanism due to the release of factors able to influence cell fate, function and plasticity. BM-MSC are a heterogeneous population of cells [40] exerting their effect also by the EVs release. For instance, they induct reduction of pro-inflammatory cytokines in graft versus host disease [32] and suppress angiogenesis in breast cancer [41]. EVs may express surface markers characteristic of originating cells [42, 43]. In fact, we found that BM-MSC derived EVs expressed several adhesion molecules of mesenchimal cells such as CD29, CD90, CD73, CD105, CD49d, CD146 and CD44. Moreover, several studies showed that EVs shuttle selected RNA proposing a new mechanism of genetic exchanging [6, 15, 44]. Sequencing BM-MSC-EVs small RNAs, we found 87 miRNAs and 5 piRNAs differentially expressed that are predicted to regulate cell differentiation and apoptosis. In particular, *miR-21* is strongly involved in apoptosis pathways [45–47]; *miR-10a* plays a crucial role in megakaryocytic differentiation [48]. Among identified piRNAs, H*sa_piR_017723_DQ594464* putatively targets *FOS*, a positive modulator of myeloid differentiation of hematopoietic progenitor [49]; *hsa_piR_020814_DQ598650* targets *SOX4*, an apoptosis regulator in different human cancers [50, 51].

BM-MSC are a component of hematopoietic microenvironment [22] and are often used with other factors (SCF and IL6) to expand UCB-CD34+ cells *in vitro* [52, 53]. UCB-CD34+ cells are an alternative source to BM and mobilized peripheral blood HSC for hematopoietic cell transplantation, in particular for patients lacking a related or an adult unrelated HLA-matched donor. The advantages of using UCB-CD34+ cells respect to the other sources are a rapid availability, absence of risk for donor and decreased incidence of acute or chronic graft versus host disease [54, 55].

In this work, for the first time, communication between the BM-MSC-EVs and the UCB-CD34+ cells was studied, demonstrating that vesicles could be useful to improve re-population of BM niche. Our data indicated that BM-MSC-EVs miRNAs and piRNAs are able to influence the fate of UCB-CD34+ cells; in fact, gene expression profile of UCB-CD34+ cells treated with BM-MSC-EVs identified about 103 up-regulated and 100 down-regulated genes respect to control. The regulation of some of these genes, as *IL6, CSF2, CCL3, CDKN1B, CEBPA, ANXA1, MPL*, was confirmed by real time-PCR data, after both EVs treatment and EVs miRNAs overexpression. Interestingly, we found a correlation between BM-MSC-EVs miRNAs/piRNAs and down-regulated genes, indicating at least one target for each EVs miRNAs/piRNAs (e.g., *CEBPA/miR-182, EGR2/miR-150* and *miR-92, MPO/ hsa_piR_020814_DQ598650*). Analyzing these genes, we identified different suppressed biological functions, as cell death and differentiation. CCAAT enhancer binding protein-a (CEBPA), for example, is one of down-regulated genes that controls the balance between cell proliferation [56, 57] and differentiation during early hematopoietic and myeloid development [58, 59]. This gene is regulated by *miR-182* [60], one of the miRNAs found by sequencing in our BM-MSC EVs. Moreover, another down-regulated gene, Early Growth Response 2 (EGR2), also involved in apoptosis [61] and differentiation [62], is regulated by two different microRNAs, identified in our sequencing data, *miR-150* [63] and *miR-92* [64]. MPO, instead, synthesized during myeloid differentiation [65, 66], is putatively regulated by our *hsa_piR_020814_DQ598650*.

All these data support a direct down-regulation by BM-MSC-EVs miRNAs/piRNAs of target genes in UCB-CD34+ cells after co-culture.

Induction of cell viability and inhibition of cellular maturation of UCB-CD34+ cells after EVs treatment respect to control were also assessed by functional tests. In particular, we observed a decreased caspase dependent apoptosis and a decreased expression of hematopoietic differentiation markers.

Moreover, we demonstrated that EVs effect is due to BM-MSC-derived EVs miRNAs. In fact, UCB-CD34+ cells, after overexpression of *miR-21-5p*, *miR-181a-5p* and *miR92a-3p* showed a significant decrease of apoptosis pathway and of caspase 3/7 activity. By contrast, over-expression of *miR-27b-3p* and *miR-10a-5p* showed a reduction of CD38 expression, i.e. a phenotypic pattern typical of undifferentiated stem cells.

Finally, gene expression profile identified 103 up-regulated genes (e.g., *IL6, CSF2, CCL3*) that, by IPA, were found under the control of miRNA targeted genes (e.g., *ZFP36/miR-27b-3p*).

Of interest, most of these genes codify for chemokines and cytokines (and their receptors) involved in the chemotaxis process of different BM cell types, thus suggesting their potential role in the reconstitution of marrow microenvironment which is fundamental for transplant engraftment. Cytokines modulate and sustain hematopoiesis acting on stem/progenitor cells and accessory cells, such as stromal cells, that can further regulate proliferation, differentiation, survival, migration and homing of these cells. Cytokine activity is mediated through specific receptors that transmit important signals for cell fate [67, 68]. CXCR4, for example, was found up-regulated in UCB after EVs treatment suggesting the induction of homing and quiescence in presence of CXCL12 [38, 39].

We demonstrated that over-expression of cytokines and chemokines after EVs treatment induces an increased migration of UCB from peripheral blood to BM in NSG mice. Of interest, *in vivo* experiments showed an improvement of UCB-CD34+ migration potential due to EVs treatment. In fact, after only 2 hours from transplantation we found halved numbers of CD34+ in peripheral blood. Our data indicated that BM-MSC-EVs could be helpful in BM microenvironment reconstitution even though the maintenance of HSCs pool over time after transplant depends on balance between self-renewal and differentiation.

In conclusion, BM-MSC-EVs combined with HSC may contribute to the hematopoietic microenvironment reconstitution representing a new therapeutic option (e.g., transplantation, gene therapy) for different diseases as hematological cancers.

## MATERIALS AND METHODS

### Cell culture

Cord blood unit were provided by Cord Blood Bank of Research Institute “Casa Sollievo della Sofferenza”, San Giovanni Rotondo. Mononuclear cells were obtained by Ficoll-Paque gradient centrifugation. UCB-CD34+ cells were isolated from mononuclear cells by CD34 Microbead Kit (Miltenyi Biotec, Auburn, CA). The purity of isolated CD34+ cells routinely ranged between 90–95%. Primary BM-MSC were isolated from bone marrow aspirate of a healthy subject provided by Prof. Francesco Frassoni's laboratory (Giannina Gaslini Institute, Genova). BM-MSC were cultured in DMEM (Gibco, Life technologies, Carlsbad, CA, USA) supplemented with 10% of fetal bovine serum (FBS) (Gibco), 1% of penicillin-streptomycin and 2 mM of L-glutamine (Gibco). Cells were grown at 37°C in 5% CO_2_. BM-MSC were seeded in 175 cm^2^ tissue culture flasks at a density of 10,000 cells/cm^2^ and used within the sixth passage of culture for experiments [42]. At each passage, cells were counted, analyzed by cytometric analysis and immunofluorescence to confirm their phenotype. Cells were characterized by FACS analysis for the expression of mesenchymal stem cell markers [69]. BM-MSC were able to undergo osteogenic, adipogenic and condrogenic differentiation when cultured in appropriate differentiative media [70].

### FACS analysis of BM-MSC cells

To confirm their phenotype, BM-MSC were analyzed by FACS Calibur cytometer using CellQuest software. Cells were labeled with following fluorochrome-conjugated monoclonal antibodies and their specific isotypic control: allophycocyanin (APC)-anti CD29 (IgG1k, Clone MAR4; BD Pharmingen); peridinin chlorophyll (PerCP)-anti CD90 (IgG1k, Clone 5E10; BD Pharmingen); phycoerythrin (PE)-anti CD73 (IgG1k, Clone AD-2; BD Pharmingen); PE-anti CD105 (IgG3, Clone 1G2, Beckman Coulter); PerCP-anti CD45 (IgG1k, Clone 2D1, BD Pharmingen); PE-anti CD49d (IgG1k, Clone 9F10; BD Pharmingen); PE-anti CD146 (IgG1k, Clone P1H12, BD Pharmingen); APC-anti CD44 (IgG2b k, Clone G44-26, BD Pharmingen); fluorescein isothiocyanate (FITC)-anti HLA-DR (IgG2a k, Clone L243, BD Pharmingen); APC-anti CD14 (IgG2b k, Clone MØP9, BD Pharmingen); PE-anti CD144 (IgG2b, Clone 123423, R & D System); FITC-anti CD31 (IgG1 k, Clone WM59, BD Pharmingen).

### Isolation and characterization of EVs

EVs were obtained from supernatants of BM-MSC cultured in DMEM supplemented with 10% FBS deprived of EVs, 1% of penicillin-streptomycin and 2 mM of L-glutamine. FBS was deprived of EVs by ultracentrifugation (Beckman Coulter, Miami, FL, USA) at 100,000 g overnight at 4°C before use.

The method used for isolation of cell derived vesicles was previously described by Théry et al. [71]. In short, BM-MSC and media were centrifuged at 300 × g for 10 min at 4°C after 48 h of culture. Supernatant was collected and centrifuged again at 2,000 × g for 20 min at 4°C; subsequently it was harvested and vacuum ultracentrifuged at 10,000 × g for 30 min at 4°C to remove residual cell debris. Supernatant was collected and ultracentrifuged at 100,000 × g for 70 min at 4°C with vacuum. The resulting supernatant was discarded; pellets from multiple tubes were resuspended in 1 ml of PBS, pooled into a single tube and ultracentrifuged at 100,000 × g as described previously. Pellets of vesicles were resuspended in filtered PBS with HEPES 25 mM and the protein content was measured by the Bradford assay (BioRad, Hercules, CA, USA). EVs were stored frozen at −80°C in DMSO 0.1%.

The size of EVs was determined by cytometric analysis. The system was calibrate using standard microbeads with a diameter of 0.3–0.9–3 μm (Megamix, BD) and prefiltered (0.22 μm) PBS was used to define EVs gate. To characterize EVs, samples were labeled using PE-CD105, PE-CD73, PE-CD146, PE-CD81 (IgG1k, Clone JS-81, BD Pharmingen), PE-CD49d, PerCP-CD45, PerCP-CD90, APC-CD29, APC-CD44 and their specific isotypic control.

### Transmission electron microscopy (TEM)

EVs from BM-MSC were suspended in PBS and loaded onto 200 mesh nickel formvar carbon coated grids (Assing, Roma). EVs were fixed in 1.25% glutaraldehyde and 1% paraformaldehyde in 0.1 M Sorensen's phosphate buffer for 15 min and then washed in 0.1 M Sorensen's phosphate buffer for 4 times. After air dry, EVs were examined by transmission electron microscopy PHILIPS CM12 TEM.

### Co-culture of UCB-CD34+ and EVs

Six million of UCB-CD34+ were plated a 6-well plate and cultured using three different conditions: a) 1.5 ml of DMEM supplemented with 5% FBS deprived of EVs, 1% penicillin-streptomycin and 2 mM L-glutamine; b) 1.5 ml of DMEM supplemented with 5% FBS deprived of EVs, 1% penicillin-streptomycin, 2 mM L-glutamine and 20 μg/ml of BM-MSC-EVs [15, 72]; c) conditioned medium of BM-MSC deprived of BM-MSC-EVs. After 24 hours of co-culture UCB-CD34^+^ cells were collected.

### Apoptosis assay

1 × 10^^5^ UCB-CD34+ treated or not with EVs were washed twice with cold PBS and then resuspended in 100 μl of 1X Binding Buffer (BD Pharmagen, Italy) with 5 μl of FITC Annexin V (BD Pharmagen, Italy) and 5 μl propidium iodide (PI) (BD Pharmagen, Italy) for 15 min at room temperature in the dark. After incubation 400 μl of 1X Binding Buffer were added to each tube and analyzed by FACSCalibur flow cytometry (BD Biosciences).

### Caspase 3/7 activity assay

Caspase 3/7 activity was measured with the CellEvent^™^ caspase 3/7 Green Detection Reagent (Life technologies). On cleavage by activated caspase 3/7, the probe becomes fluorescent and free to bind to DNA. Cells were incubated with 1 μM CellEvent^™^ caspase 3/7 green detection reagent in complete medium for 30 min at 37°C in the dark. Stained cells were observed by flow cytometry.

### RNA extraction

RNA was extracted using Trizol reagent (Life Technologies) according to the manufacturer's instructions. Total RNA was quantified with a NanoDrop 2000c spectrophotometer (Thermo Scientific, Wilmington, DE, USA) and its quality was assessed by capillary electrophoresis on an Agilent 2100 Bioanalyzer (Agilent Technologies, Inc, Santa Clara, CA) using RNA 6000 Nano or RNA Pico Chip Assay Kit (Agilent). The presence of small RNAs in EVs samples was verified using Small RNA Chip (Agilent). For RNA isolated from UCB-CD34 + cells, only a RNA integrity number (RIN) > 8 was used. Since the intact 18S and 28S rRNA were scarcely detectable in EVs, the RIN was not a constrain for these samples.

### Microarray data analysis

For each sample, 300 ng of total RNA was reverse transcribed and used for synthesis of cDNA and biotinylated cRNA according to the Illumina TotalPrep RNA amplification kit protocol (Ambion, Austin, TX; category number IL1791). Hybridization of 750 ng of cRNA on Illumina HumanHT12 v4.0 Expression BeadChip array (Illumina Inc., San Diego, CA, USA), staining and scanning were performed according to the standard protocol supplied by Illumina. All preparation was performed in triplicate for each sample. BeadChip was dried and scanned with an Illumina HiScanSQ system (Illumina Inc.).

The intensity files were loaded into the Illumina Genome Studio software for quality control and gene expression analysis. Quantile normalization algorithm was applied on the data set to correct systematic errors, values below a detection score of 0.05 were filtered out and missing values were imputed. Differently expressed genes (DEGs) were selected with differential score (DiffScore) cutoff set at ± 30 (*p* < 0.001) and genes with log2fold difference ≤ −0.7 or ≥ 0.7 (more than 1.5-fold change) were further analyzed (Supplementary Table 2). Microarray data were submitted to Array Express under accession number E-MTAB-3576. DEGs list (composed by 230 genes, 103 up-regulated and 100 down-regulated) was used to evaluate the functional behavior in terms of Biological Processes performing an enrichment analysis with Ingenuity Pathway Analysis (IPA) (Ingenuity Systems; Mountain View, CA, USA).

### Small RNA sequencing

For sequencing, 1 μg of total RNA/EVs pool was used for library preparation with Illumina TruSeq small RNA sample preparation Kit. The library (10 pM) was sequenced on HiSeq1000 (Illumina) for 50 cycles. Small RNA sequencing data were analyzed using iMir [33] and mature miRNA list obtained with miRanalyzer [73] was used to characterize EVs small RNA content. Mature microRNAs with a readCount less than 5 were removed. Final mature microRNAs readCount list was subdivided in quartiles on readCount number (Supplementary Table 1) and only the 1st was used in the integrated analysis with gene expression profiling array (Table 1).

The sequencing data were preprocessed for piRNAs by Flexbar v.2.5 [74] and then analyzed by piPipes smallRNA v. 1.0.2. [34] (piRNA length range: 23–36 nt). PiRNAs with a readCount less than 5 were removed (Figure 2F).

The most representative sequences were classified in piRNABank (Institute of Bioinformatics and Applied Biotechnology, Bangalore, India) [75] and putative piRNA target RNAs were identified using miRanda v.3.3a [35] with alignment score (sc; ≥ 170) and energy threshold (en; ≤ −20.0 kcal/mol) by complementary sequence between each piRNA and the 5′-UTRs, CDSs or 3′-UTRs of all known human RNAs (Ensembl gene annotation). The piRNA gene list was crossed with gene expression profile identifying 18 common piRNA target genes used then for integrated analysis with IPA (*p*-value < 0.05 and logFC ≤ −0.4).

Raw small RNA sequencing data are available in NCBI Gene Expression Omnibus (GEO) database (http://www.ncbi.nlm.nih.gov/gds/) with Accession Number SRP058423.

### Quantitative real-time PCR

Quantitative real-time PCRs (qRT-PCRs) were performed with the TaqMan PCR Kit (Applied Biosystems, Foster City, CA) according to manufacturer's instructions, followed by detection with the Light Cycler 480 (Roche, Australia). The appropriate TaqMan probes for mRNA and microRNA quantification were purchased from Applied Biosystems. All reactions were performed in triplicate. Simultaneous quantification of *GAPDH* mRNAs and *RNU44* were used as a reference for mRNA and microRNA TaqMan assay data normalization. The comparative cycle threshold (Ct) method for relative quantification of gene expression (User Bulletin #2; Applied Biosystems) was used to determine mRNA expression levels.

### UCB-CD34+ cell transfection

UCB-CD34+ were transfected with 60 nM of miRNA precursor molecules (*miR-27b-3p* mimic, *miR-10a-5p* mimic, *miR-21-5p* mimic, *miR-181a-5p* mimic and *miR92a-3p* mimic) (Life Technologies) or negative control (Life Technologies) using Lipofectamine 2000 (Life Technologies), according to the manifacturer's instructions. After 24 hours of transfection cells were collected and used for cytometric analysis and for RNA isolation.

### Mice

For *in vivo* studies, 8-week old NOD-SCID-Il2rg−/− (NSG) mice were purchased by Jackson Laboratory. The experimental protocol was approved by the Institutional Animal Facility of Biogem Research Institute. 2 × 10^5^ UCB-CD34+ cells treated or not with EVs for 24 hours were injected by tail vein into every mouse. After 2 and 24 hours from injection, sub-mandibular blood was collected. For analysis of engraftment experimental mice were sacrificed 24 hours post-transplantation and the BM was obtained from femur and tibia. To determine the number of CD34+/μl, blood was transferred in truCOUNT tube, incubated with specific anti-human CD45 FITC/CD34 PE, lysed with 1X ammonium chloride lysing solution according to the manufacturer's procedures (BD Stem Cell Enumeration Kit) and acquired by FACS CANTO II. Analysis was performed with FACS DIVA software. CD34 + number was calculated with the formula CD34+/μl = CD34 + acquired/ trucount events × total bead count/100 μl of blood.

Cells from BM were labeled with specific anti-human CD45 FITC/CD34 PE and resuspended in PBS. For each sample 5 × 10^6^ events were acquired. CD34+ rate was expressed as number of positive cells/million of acquired events.

### Statistical analysis

Student's *t*-test was used to determine significance (indicated as *p*-value). All error bars represent the standard deviation (SD) of the mean.

### IPA and integrated analysis

Gene interaction networks, bio functions and pathway analysis were generated by IPA, which assists with microarray data interpretation via grouping differentially expressed genes (DEGs) into known functions, pathways, and networks primarily based on human and rodent studies. The DEGs were organized in Gene Ontology Bio Functions and Regulatory Effect Networks available from the Ingenuity database. The significance was set at a *p*-value of 0.05.

IPA pathway explorer and TargetScan (http://www.targetscan.org/) were used to find a link between down-regulated genes in array experiment and 1st quartile of miRNA sequenced by EVs, looking for putative causal effect in EVs treatment of UCB-CD34+.

## SUPPLEMENTARY MATERIALS TABLES


