# Supplementary material for: MiRNAs and piRNAs from bone marrow mesenchymal stem cell extracellular vesicles induce cell survival and inhibit cell differentiation of cord blood hematopoietic stem cells: a new insight in transplantation
Source: Oncotarget. 2015 Dec 29;7(6):6676–92. doi: 10.18632/oncotarget.6791 (PMC4872742; doi:10.18632/oncotarget.6791)
Supplement: Supplementary file 1 [file oncotarget-07-6676-s001.pdf]

MiRNAs and piRNAs from bone marrow mesenchymal stem cell extracellular vesicles induce cell survival and inhibit cell differentiation of cord blood hematopoietic stem cells: a new insight in transplantation

Supplementary Materials

Supplementary Table S1: Mature EVs miRNAs analyzed with small RNA sequencing

Quartile	hsa-miR name	readCount	#uniqueReads	norm_expressed_all	norm_expressed_mapped
1st quartile	**hsa-miR-3168**	14423	65	9.04E–01	6.30E + 01
	**hsa-miR-22-3p**	2447	22	1.53E–01	1.07E + 01
	**hsa-miR-378a-3p**	775	15	4.86E–02	3.38E + 00
	**hsa-miR-205-5p**	523	15	3.28E–02	2.28E + 00
	**hsa-miR-203a**	515	20	3.23E–02	2.25E + 00
	**hsa-miR-27b-3p**	458	15	2.87E–02	2.00E + 00
	**hsa-miR-423-5p**	347	14	2.17E–02	1.52E + 00
	**hsa-miR-21-5p**	272	14	1.70E–02	1.19E + 00
	**hsa-miR-181a-5p**	231	12	1.45E–02	1.01E + 00
	**hsa-miR-92a-3p**	221	14	1.38E–02	9.65E – 01
	**hsa-miR-146b-5p**	161	10	1.01E–02	7.03E – 01
	**hsa-miR-10a-5p**	114	7	7.14E–03	4.98E – 01
	**hsa-miR-26a-5p**	112	6	7.02E–03	4.89E – 01
	**hsa-miR-148a-3p**	107	8	6.70E–03	4.67E – 01
	**hsa-let-7f-5p**	102	5	6.39E–03	4.45E – 01
	**hsa-miR-486-5p**	98	10	6.14E–03	4.28E – 01
	**hsa-miR-28-3p**	96	10	6.02E–03	4.19E – 01
	**hsa-miR-4792**	92	7	5.76E–03	4.02E – 01
	**hsa-let-7a-5p**	84	5	5.26E–03	3.67E – 01
	**hsa-miR-3182**	83	3	5.20E–03	3.62E – 01
	**hsa-miR-423-3p**	80	6	5.01E–03	3.49E – 01
2nd quartile	**hsa-miR-4516**	70	8	4.39E–03	3.06E – 01
	**hsa-miR-1246**	69	8	4.32E–03	3.01E – 01
	**hsa-miR-222-3p**	61	4	3.82E–03	2.66E – 01
	**hsa-miR-320a**	59	11	3.70E–03	2.58E – 01
	**hsa-let-7i-5p**	53	5	3.32E–03	2.31E – 01
	**hsa-miR-4301**	53	5	3.32E–03	2.31E – 01
	**hsa-miR-21-3p**	50	4	3.13E–03	2.18E – 01
	**hsa-miR-191-5p**	46	5	2.88E–03	2.01E – 01
	**hsa-let-7b-5p**	44	10	2.76E–03	1.92E – 01
	**hsa-miR-4448**	36	3	2.26E–03	1.57E – 01
	**hsa-miR-127-3p**	35	4	2.19E–03	1.53E – 01
	**hsa-miR-92b-3p**	33	4	2.07E–03	1.44E – 01
	**hsa-miR-182-5p**	32	5	2.01E–03	1.40E – 01
	**hsa-miR-1307-5p**	31	5	1.94E–03	1.35E – 01
	**hsa-miR-192-5p**	27	3	1.69E–03	1.18E – 01
	**hsa-miR-146a-5p**	26	4	1.63E–03	1.14E – 01
	**hsa-miR-210-3p**	23	5	1.44E–03	1.00E – 01
	**hsa-miR-221-3p**	23	3	1.44E–03	1.00E – 01
	**hsa-miR-25-3p**	21	1	1.32E–03	9.17E – 02
	**hsa-miR-4488**	20	5	1.25E–03	8.73E – 02
	**hsa-miR-432-5p**	20	4	1.25E–03	8.73E – 02
	**hsa-miR-30a-5p**	20	2	1.25E–03	8.73E – 02

3rd quartile	**hsa-miR-769-5p**	19	1	1.19E–03	8.30E – 02
	**hsa-miR-30e-5p**	17	1	1.07E–03	7.42E – 02
	**hsa-miR-151a-3p**	16	5	1.00E–03	6.99E – 02
	**hsa-miR-181b-5p**	15	3	9.40E–04	6.55E – 02
	**hsa-let-7e-5p**	15	3	9.40E–04	6.55E – 02
	**hsa-miR-30d-5p**	14	4	8.77E–04	6.11E – 02
	**hsa-miR-103a-3p**	14	1	8.77E–04	6.11E – 02
	**hsa-miR-181a-2-3p**	12	3	7.52E–04	5.24E – 02
	**hsa-miR-30c-5p**	12	2	7.52E–04	5.24E – 02
	**hsa-miR-532-5p**	12	2	7.52E–04	5.24E – 02
	**hsa-let-7g-5p**	10	3	6.27E–04	4.37E – 02
	**hsa-miR-142-5p**	10	3	6.27E–04	4.37E – 02
	**hsa-miR-27a-5p**	10	2	6.27E–04	4.37E – 02
	**hsa-miR-654-3p**	10	1	6.27E–04	4.37E – 02
	**hsa-miR-3960**	9	4	5.64E–04	3.93E – 02
	**hsa-miR-141-3p**	9	2	5.64E–04	3.93E – 02
	**hsa-miR-27a-3p**	9	2	5.64E–04	3.93E – 02
	**hsa-miR-223-3p**	9	1	5.64E–04	3.93E – 02
	**hsa-miR-186-5p**	8	3	5.01E–04	3.49E – 02
	**hsa-miR-877-5p**	8	3	5.01E–04	3.49E – 02
	**hsa-miR-3615**	8	2	5.01E–04	3.49E – 02
	**hsa-miR-27b-5p**	8	1	5.01E–04	3.49E – 02
	**hsa-miR-4532**	7	2	4.39E–04	3.06E – 02
	**hsa-miR-26b-5p**	7	2	4.39E–04	3.06E – 02
	**hsa-miR-125a-5p**	7	1	4.39E–04	3.06E – 02
4th quartile	**hsa-miR-212-3p**	7	1	4.39E–04	3.06E – 02
	**hsa-miR-125b-1-3p**	6	2	3.76E–04	2.62E – 02
	**hsa-miR-100-5p**	6	2	3.76E–04	2.62E – 02
	**hsa-miR-150-5p**	6	2	3.76E–04	2.62E – 02
	**hsa-miR-148b-3p**	6	2	3.76E–04	2.62E – 02
	**hsa-miR-339-3p**	6	2	3.76E–04	2.62E – 02
	**hsa-miR-93-3p**	6	1	3.76E–04	2.62E – 02
	**hsa-miR-212-5p**	6	1	3.76E–04	2.62E – 02
	**hsa-miR-1468-5p**	6	1	3.76E–04	2.62E – 02
	**hsa-miR-574-5p**	6	1	3.76E–04	2.62E – 02
	**hsa-miR-7641**	5	2	3.13E–04	2.18E – 02
	**hsa-miR-31-5p**	5	2	3.13E–04	2.18E – 02
	**hsa-miR-345-5p**	5	2	3.13E–04	2.18E – 02
	**hsa-miR-16-5p**	5	2	3.13E–04	2.18E – 02
	**hsa-miR-101-3p**	5	2	3.13E–04	2.18E – 02
	**hsa-miR-130a-3p**	5	1	3.13E–04	2.18E – 02
	**hsa-miR-205-3p**	5	1	3.13E–04	2.18E – 02
	**hsa-miR-193b-5p**	5	1	3.13E–04	2.18E – 02
	**hsa-miR-5096**	5	1	3.13E–04	2.18E – 02

Supplementary Table S2: Differentially expressed genes in UCB-CD34+ treated with EVs vs control (*p*-value < 0.05, logFC = 0.7)

ID Entrez	Symbol	Entrez Gene Name	Log Ratio
**3569**	IL6	interleukin 6	4.396
**6346**	CCL1	chemokine (C-C motif) ligand 1	3.456
**1437**	CSF2	colony stimulating factor 2 (granulocyte-macrophage)	3.151
**10563**	CXCL13	chemokine (C-X-C motif) ligand 13	3.086
**10148**	EBI3	Epstein-Barr virus induced 3	2.884
**5743**	PTGS2	prostaglandin-endoperoxide synthase 2 (prostaglandin G/H synthase and cyclooxygenase)	2.696
**6363**	CCL19	chemokine (C-C motif) ligand 19	2.66
**56300**	IL36G	interleukin 36, gamma	2.541
**6364**	CCL20	chemokine (C-C motif) ligand 20	2.144
**6349**	CCL3L1	chemokine (C-C motif) ligand 3-like 1	2.124
**83862**	TMEM120A	transmembrane protein 120A	2.065
**6348**	CCL3	chemokine (C-C motif) ligand 3	2.034
**10537**	UBD	ubiquitin D	1.953
**6372**	CXCL6	chemokine (C-X-C motif) ligand 6	1.941
**3553**	IL1B	interleukin 1, beta	1.933
**3627**	CXCL10	chemokine (C-X-C motif) ligand 10	1.877
**3488**	IGFBP5	insulin-like growth factor binding protein 5	1.876
**2920**	CXCL2	chemokine (C-X-C motif) ligand 2	1.821
**9536**	PTGES	prostaglandin E synthase	1.778
**3620**	IDO1	indoleamine 2,3-dioxygenase 1	1.656
**3779**	KCNMB1	potassium large conductance calcium-activated channel, subfamily M, beta member 1	1.624
**7130**	TNFAIP6	tumor necrosis factor, alpha-induced protein 6	1.61
**3552**	IL1A	interleukin 1, alpha	1.584
**4502**	MT2A	metallothionein 2A	1.572
**6648**	SOD2	superoxide dismutase 2, mitochondrial	1.547
**2919**	CXCL1	chemokine (C-X-C motif) ligand 1	1.45
**57817**	HAMP	hepcidin antimicrobial peptide	1.426
**4495**	MT1G	metallothionein 1G	1.424
**8710**	SERPINB7	serpin peptidase inhibitor, clade B (ovalbumin), member 7	1.416
**4489**	MT1A	metallothionein 1A	1.407
**114614**	MIR155HG	MIR155 host gene (non-protein coding)	1.394
**11009**	IL24	interleukin 24	1.389
**5327**	PLAT	plasminogen activator, tissue	1.387
**2633**	GBP1	guanylate binding protein 1, interferon-inducible	1.348
**1236**	CCR7	chemokine (C-C motif) receptor 7	1.279
**6775**	STAT4	signal transducer and activator of transcription 4	1.279
**4790**	NFKB1	nuclear factor of kappa light polypeptide gene enhancer in B-cells 1	1.277
**6890**	TAP1	transporter 1, ATP-binding cassette, sub-family B (MDR/TAP)	1.236

**3669**	ISG20	interferon stimulated exonuclease gene 20kDa	1.235
**1462**	VCAN	versican	1.226
**22846**	VASH1	vasohibin 1	1.219
**94235**	GNG8	guanine nucleotide binding protein (G protein), gamma 8	1.211
**5996**	RGS1	regulator of G-protein signaling 1	1.194
**5553**	PRG2	proteoglycan 2, bone marrow (natural killer cell activator, eosinophil granule major basic protein)	1.186
**8809**	IL18R1	interleukin 18 receptor 1	1.185
**6367**	CCL22	chemokine (C-C motif) ligand 22	1.181
**114897**	C1QTNF1	C1q and tumor necrosis factor related protein 1	1.149
**8743**	TNFSF10	tumor necrosis factor (ligand) superfamily, member 10	1.144
**9662**	CEP135	centrosomal protein 135kDa	1.131
**5366**	PMAIP1	phorbol-12-myristate-13-acetate-induced protein 1	1.107
**145864**	HAPLN3	hyaluronan and proteoglycan link protein 3	1.1
**9367**	RAB9A	RAB9A, member RAS oncogene family	1.07
**4499**	MT1M	metallothionein 1M	1.067
**64092**	SAMSN1	SAM domain, SH3 domain and nuclear localization signals 1	1.065
**7292**	TNFSF4	tumor necrosis factor (ligand) superfamily, member 4	1.065
**11001**	SLC27A2	solute carrier family 27 (fatty acid transporter), member 2	1.053
**115361**	GBP4	guanylate binding protein 4	1.033
**8870**	IER3	immediate early response 3	1.029
**23180**	RFTN1	raftlin, lipid raft linker 1	1.022
**3429**	IFI27	interferon, alpha-inducible protein 27	1.021
**3559**	IL2RA	interleukin 2 receptor, alpha	1.016
**57801**	HES4	hes family bHLH transcription factor 4	1.015
**7453**	WARS	tryptophanyl-tRNA synthetase	0.993
**3589**	IL11	interleukin 11	0.989
**135**	ADORA2A	adenosine A2a receptor	0.983
**2532**	ACKR1	atypical chemokine receptor 1 (Duffy blood group)	0.971
**10135**	NAMPT	nicotinamide phosphoribosyltransferase	0.97
**10538**	BATF	basic leucine zipper transcription factor, ATF-like	0.967
**11040**	PIM2	Pim-2 proto-oncogene, serine/threonine kinase	0.96
**6624**	FSCN1	fascin actin-bundling protein 1	0.956
**4885**	NPTX2	neuronal pentraxin II	0.953
**51312**	SLC25A37	solute carrier family 25 (mitochondrial iron transporter), member 37	0.952
**56265**	CPXM1	carboxypeptidase X (M14 family), member 1	0.951
**115362**	GBP5	guanylate binding protein 5	0.947
**388372**	CCL4L1/ CCL4L2	chemokine (C-C motif) ligand 4-like 1	0.931
**3456**	IFNB1	interferon, beta 1, fibroblast	0.93
**11274**	USP18	ubiquitin specific peptidase 18	0.93
**9173**	IL1RL1	interleukin 1 receptor-like 1	0.927
**9638**	FEZ1	fasciculation and elongation protein zeta 1 (zygin I)	0.926
**51561**	IL23A	interleukin 23, alpha subunit p19	0.923

**306**	ANXA3	annexin A3	0.909
**2706**	GJB2	gap junction protein, beta 2, 26kDa	0.908
**9242**	MSC	musculin	0.9
**7295**	TXN	thioredoxin	0.897
**8140**	SLC7A5	solute carrier family 7 (amino acid transporter light chain, L system), member 5	0.895
**8738**	CRADD	CASP2 and RIPK1 domain containing adaptor with death domain	0.891
**10409**	BASP1	brain abundant, membrane attached signal protein 1	0.881
**64218**	SEMA4A	sema domain, immunoglobulin domain (Ig), transmembrane domain (TM) and short cytoplasmic domain, (semaphorin) 4A	0.879
**57007**	ACKR3	atypical chemokine receptor 3	0.869
**139411**	PTCHD1	patched domain containing 1	0.862
**4791**	NFKB2	nuclear factor of kappa light polypeptide gene enhancer in B-cells 2 (p49/p100)	0.856
**11182**	SLC2A6	solute carrier family 2 (facilitated glucose transporter), member 6	0.855
**5209**	PFKFB3	6-phosphofructo-2-kinase/fructose-2,6-biphosphatase 3	0.843
**4049**	LTA	lymphotoxin alpha	0.829
**4001**	LMNB1	lamin B1	0.818
**23413**	NCS1	neuronal calcium sensor 1	0.818
**5008**	OSM	oncostatin M	0.814
**890**	CCNA2	cyclin A2	0.801
**3576**	CXCL8	chemokine (C-X-C motif) ligand 8	0.8
**375449**	MAST4	microtubule associated serine/threonine kinase family member 4	0.792
**83641**	FAM107B	family with sequence similarity 107, member B	0.792
**23753**	SDF2L1	stromal cell-derived factor 2-like 1	0.787
**3604**	TNFRSF9	tumor necrosis factor receptor superfamily, member 9	0.787
**10938**	EHD1	EH-domain containing 1	0.784
**127544**	RNF19B	ring finger protein 19B	0.774
**970**	CD70	CD70 molecule	0.765
**644314**	MT1IP	metallothionein 1I, pseudogene	0.764
**1847**	DUSP5	dual specificity phosphatase 5	0.762
**55848**	PLGRKT	plasminogen receptor, C-terminal lysine transmembrane protein	0.762
**135112**	NCOA7	nuclear receptor coactivator 7	0.761
**9833**	MELK	maternal embryonic leucine zipper kinase	0.759
**10410**	IFITM3	interferon induced transmembrane protein 3	0.754
**6373**	CXCL11	chemokine (C-X-C motif) ligand 11	0.754
**22875**	ENPP4	ectonucleotide pyrophosphatase/phosphodiesterase 4 (putative)	0.752
**29957**	SLC25A24	solute carrier family 25 (mitochondrial carrier; phosphate carrier), member 24	0.751
**654**	BMP6	bone morphogenetic protein 6	0.739
**51316**	PLAC8	placenta-specific 8	0.739
**8651**	SOCS1	suppressor of cytokine signaling 1	0.737
**9595**	CYTIP	cytohesin 1 interacting protein	0.734
**7128**	TNFAIP3	tumor necrosis factor, alpha-induced protein 3	0.734
**2117**	ETV3	ets variant 3	0.728
**4494**	MT1F	metallothionein 1F	0.724

**25805**	BAMBI	BMP and activin membrane-bound inhibitor	0.723
**4091**	SMAD6	SMAD family member 6	0.716
**8544**	PIR	pirin (iron-binding nuclear protein)	0.713
**952**	CD38	CD38 molecule	0.707
**4312**	MMP1	matrix metallopeptidase 1 (interstitial collagenase)	0.703
**5698**	PSMB9	proteasome (prosome, macropain) subunit, beta type, 9	0.703
**4493**	MT1E	metallothionein 1E	0.701
**64108**	RTP4	receptor (chemosensory) transporter protein 4	0.7
**56603**	CYP26B1	cytochrome P450, family 26, subfamily B, polypeptide 1	−0.701
**9920**	KBTBD11	kelch repeat and BTB (POZ) domain containing 11	−0.702
**55357**	TBC1D2	TBC1 domain family, member 2	−0.708
**30061**	SLC40A1	solute carrier family 40 (iron-regulated transporter), member 1	−0.711
**23643**	LY96	lymphocyte antigen 96	−0.712
**8854**	ALDH1A2	aldehyde dehydrogenase 1 family, member A2	−0.712
**166012**	CHST13	carbohydrate (chondroitin 4) sulfotransferase 13	−0.717
**284422**	SMIM24	small integral membrane protein 24	−0.721
**440712**	C1orf186	chromosome 1 open reading frame 186	−0.722
**3490**	IGFBP7	insulin-like growth factor binding protein 7	−0.724
**23646**	PLD3	phospholipase D family, member 3	−0.725
**4048**	LTA4H	leukotriene A4 hydrolase	−0.735
**2354**	FOSB	FBJ murine osteosarcoma viral oncogene homolog B	−0.737
**2208**	FCER2	Fc fragment of IgE, low affinity II, receptor for (CD23)	−0.74
**6037**	RNASE3	ribonuclease, RNase A family, 3	−0.742
**222166**	MTURN	maturin, neural progenitor differentiation regulator homolog (Xenopus)	−0.742
**1848**	DUSP6	dual specificity phosphatase 6	−0.743
**11098**	PRSS23	protease, serine, 23	−0.745
**1393**	CRHBP	corticotropin releasing hormone binding protein	−0.747
**847**	CAT	catalase	−0.752
**10870**	HCST	hematopoietic cell signal transducer	−0.758
**442582**	STAG3L2/ STAG3L3	stromal antigen 3-like 3	−0.758
**1027**	CDKN1B	cyclin-dependent kinase inhibitor 1B (p27, Kip1)	−0.764
**51738**	GHRL	ghrelin/obestatin prepropeptide	−0.766
**11010**	GLIPR1	GLI pathogenesis-related 1	−0.77
**56833**	SLAMF8	SLAM family member 8	−0.771
**6036**	RNASE2	ribonuclease, RNase A family, 2 (liver, eosinophil-derived neurotoxin)	−0.775
**645638**	WFDC21P	WAP four-disulfide core domain 21, pseudogene	−0.779
**81501**	DCSTAMP	dendrocyte expressed seven transmembrane protein	−0.779
**3202**	HOXA5	homeobox A5	−0.788
**3045**	HBD	hemoglobin, delta	−0.788
**5997**	RGS2	regulator of G-protein signaling 2	−0.788
**10365**	KLF2	Kruppel-like factor 2	−0.79
**6518**	SLC2A5	solute carrier family 2 (facilitated glucose/fructose transporter), member 5	−0.794

**6503**	SLA	Src-like-adaptor	−0.802
**284**	ANGPT1	angiopoietin 1	−0.802
**10184**	LHFPL2	lipoma HMGIC fusion partner-like 2	−0.806
**84803**	AGPAT9	1-acylglycerol-3-phosphate O-acyltransferase 9	−0.81
**10261**	IGSF6	immunoglobulin superfamily, member 6	−0.817
**6281**	S100A10	S100 calcium binding protein A10	−0.818
**6386**	SDCBP	syndecan binding protein (syntenin)	−0.82
**1992**	SERPINB1	serpin peptidase inhibitor, clade B (ovalbumin), member 1	−0.823
**137075**	CLDN23	claudin 23	−0.839
**2745**	GLRX	glutaredoxin (thioltransferase)	−0.84
**79026**	AHNAK	AHNAK nucleoprotein	−0.843
**4071**	TM4SF1	transmembrane 4 L six family member 1	−0.867
**1991**	ELANE	elastase, neutrophil expressed	−0.869
**23601**	CLEC5A	C-type lectin domain family 5, member A	−0.872
**9332**	CD163	CD163 molecule	−0.878
**2359**	FPR3	formyl peptide receptor 3	−0.884
**3205**	HOXA9	homeobox A9	−0.889
**9834**	KIAA0125	KIAA0125	−0.89
**4613**	MYCN	v-myc avian myelocytomatosis viral oncogene neuroblastoma derived homolog	−0.894
**64798**	DEPTOR	DEP domain containing MTOR-interacting protein	−0.899
**8334**	HIST1H2AC	histone cluster 1, H2ac	−0.899
**10577**	NPC2	Niemann-Pick disease, type C2	−0.9
**123**	PLIN2	perilipin 2	−0.926
**2205**	FCER1A	Fc fragment of IgE, high affinity I, receptor for; alpha polypeptide	−0.929
**2124**	EVI2B	ecotropic viral integration site 2B	−0.947
**1230**	CCR1	chemokine (C-C motif) receptor 1	−0.971
**10437**	IFI30	interferon, gamma-inducible protein 30	−0.994
**10077**	TSPAN32	tetraspanin 32	−1.001
**3988**	LIPA	lipase A, lysosomal acid, cholesterol esterase	−1.022
**7305**	TYROBP	TYRO protein tyrosine kinase binding protein	−1.025
**55803**	ADAP2	ArfGAP with dual PH domains 2	−1.03
**2268**	FGR	FGR proto-oncogene, Src family tyrosine kinase	−1.042
**7941**	PLA2G7	phospholipase A2, group VII (platelet-activating factor acetylhydrolase, plasma)	−1.072
**678**	ZFP36L2	ZFP36 ring finger protein-like 2	−1.072
**27128**	CYTH4	cytohesin 4	−1.075
**11027**	LILRA2	leukocyte immunoglobulin-like receptor, subfamily A (with TM domain), member 2	−1.075
**4973**	OLR1	oxidized low density lipoprotein (lectin-like) receptor 1	−1.082
**54360**	CYTL1	cytokine-like 1	−1.115
**1178**	CLC	Charcot-Leyden crystal galectin	−1.144
**10457**	GPNMB	glycoprotein (transmembrane) nmb	−1.179
**1050**	CEBPA	CCAAT/enhancer binding protein (C/EBP), alpha	−1.182
**255631**	COL24A1	collagen, type XXIV, alpha 1	−1.23

**1593**	CYP27A1	cytochrome P450, family 27, subfamily A, polypeptide 1	−1.241
**6446**	SGK1	serum/glucocorticoid regulated kinase 1	−1.272
**1536**	CYBB	cytochrome b-245, beta polypeptide	−1.302
**199675**	MCEMP1	mast cell-expressed membrane protein 1	−1.308
**4352**	MPL	MPL proto-oncogene, thrombopoietin receptor	−1.329
**301**	ANXA1	annexin A1	−1.391
**25907**	TMEM158	transmembrane protein 158 (gene/pseudogene)	−1.393
**7045**	TGFBI	transforming growth factor, beta-induced, 68kDa	−1.397
**6691**	SPINK2	serine peptidase inhibitor, Kazal type 2	−1.434
**1545**	CYP1B1	cytochrome P450, family 1, subfamily B, polypeptide 1	−1.448
**54**	ACP5	acid phosphatase 5, tartrate resistant	−1.492
**4318**	MMP9	matrix metallopeptidase 9 (gelatinase B, 92kDa gelatinase, 92kDa type IV collagenase)	−1.518
**1436**	CSF1R	colony stimulating factor 1 receptor	−1.55
**28984**	RGCC	regulator of cell cycle	−1.634
**1514**	CTSL	cathepsin L	−1.657
**2012**	EMP1	epithelial membrane protein 1	−1.669
**1043**	CD52	CD52 molecule	−1.747
**1959**	EGR2	early growth response 2	−1.8
**4353**	MPO	myeloperoxidase	−2.024
**4069**	LYZ	lysozyme	−2.071
**6354**	CCL7	chemokine (C-C motif) ligand 7	−2.085
**5473**	PPBP	pro-platelet basic protein	−2.468
**2203**	FBP1	fructose-1,6-bisphosphatase 1	−2.989
**6369**	CCL24	chemokine (C-C motif) ligand 24	−3.003

Supplementary Table S3: Correlation of sequenced EVs piRNAs and down-regulated target genes in UCB-Cd34+ treated with BM-MSC-EVs (*p*-value < 0.05, logFC = −0.4)

miRNA		Regulated genes
Name	***readCount***	***Symbol (Entrez Gene ID)***
**hsa_piR_004307_DQ575881**	2965.67	**FES** (2242)
**hsa_piR_017723_DQ594464**	643.333	**FOS** (2353), **HNRNPA0** (10949), **RABGAP1** (23637)
**hsa_piR_020814_DQ598650**	13	**CDK6** (1021), **HBG2** (3048), **MAP3K1** (4214), **MPO** (4353), **RYR3** (6263), **SLAMF8** (56833), **SLC2A5** (6518), **SOX4** (6659), **STOM** (2040), **TPP1** (1200), **YPEL5** (51646)
**hsa_piR_016745_DQ593052**	11.6667	**ANKRD28** (23243)
**hsa_piR_002732_DQ573682**	6	**CYP1B1** (1545), **USP3** (1545)

Supplementary Table S4: Molecular and cellular functions of down-regulated piRNAs targeted genes (p-value < 0.05, logFC = −0.4)

Gene Ontology Bio Functions	p-value
Molecular and Cellular Functions	
**Cell Morphology**	3.28^{-02}–3.54^{-05}
**Cellular Compromise**	2.95^{-02}–4.35^{-04}
**Cell Death and Survival**	3.12^{-02}–4.85^{-04}
**Cellular Development**	3.12^{-02}–5.01^{-04}
**Cellular Growth and Proliferation**	2.95^{-02}–5.01^{-04}
